# Provider costs of Antiretroviral therapy (ART) in Zimbabwe: The value of using time-driven activity based costing methods in a low resource setting

**DOI:** 10.1371/journal.pone.0345316

**Published:** 2026-03-25

**Authors:** Juliet Gamuchirai Nyamasve, Carren Pindiriri, Tonderai Mapako, Alex Ingwani, Shevone Corbin, Owen Mugurungi, Tsitsi Apollo, Shepherd Chikomo, Kundai Chikambure, Shepherd Shamu, Linden Morrison, Nokuthula Mujuru, Emmanuel Boadi, Tatjana Peterson, Joconiah Chirenda

**Affiliations:** 1 Department of Global, Public Health and Family Medicine, University of Zimbabwe, Harare, Zimbabwe; 2 Department of Economics and Development, University of Zimbabwe, Harare, Zimbabwe; 3 National Blood Service Zimbabwe, Harare, Zimbabwe; 4 AIDS and TB Programme, Ministry of Health and Child Care, Harare, Zimbabwe; 5 Global Fund, Global Health Campus, Geneva, Switzerland; 6 United Nations Development Programme Zimbabwe, Harare, Zimbabwe; University of Zimbabwe, Faculty of Medicine and Health Sciences, ZIMBABWE

## Abstract

Although ART has transformed HIV into a manageable chronic condition, significant cost and logistical challenges persist, threatening progress toward the UNAIDS 95-95-95 targets. Budget allocation to the health sector declined by over 30% in the last decade in Zimbabwe, attributed to donor fatigue and emergence of pandemics. The time-driven activity-based costing (TDABC) method was used to estimate the provider costs of ART and inform resource allocation for sustained ART programming. A descriptive cross-sectional study in 11 facilities across Zimbabwe’s four levels of care collected data using standardized instruments, capturing over 2,500 provider-recipient observations. Process maps of HIV care pathways were developed with subject matter experts to document resource use and standard of care. Time taken to deliver ART services, cost of space and cost of equipment were used to calculate costs and validated by national level stakeholders. In 2022, annual provider costs for ART in totalled $168.66 million for 1.2 million patients. National costs are projected to $192.44 million by 2026, attributed to declining HIV-related mortality and incidence. Primary care facilities bore 75% of costs due to higher patient volume. Provider costs averaged $57.05 for adult ART initiation and $62.70 for paediatric initiation. First-year ART costs per client were $252.78 (adult) and $450.56 (paediatric). Annual maintenance costs were $138.93 for first-line and $174.93 for second-line ART. Laboratory services ($30.72) contributed more to adult ART costs than medicines ($27.98). ART costs exceeded prior estimates, driven by facility-level differences, laboratory expenses, and paediatric formulations. Task-shifting proved cost-efficient, but sustainability is threatened by funding gaps and low health worker compensation. Optimizing laboratory systems and decentralizing services remain critical. External funding withdrawal created an annual gap of more than $50 million. Sustaining ART to 2030, requires improving domestic resource mobilization, strengthening ART decentralization, and designing cost-efficient laboratory models that preserve treatment quality.

## Introduction

Globally, HIV infection has long been a pressing public health concern with over 39 million recorded cases [1]. Sub-Saharan Africa represents the epidemic’s epicenter, housing over two-thirds of all HIV cases, with women and girls disproportionately affected. The HIV treatment landscape has transitioned from a global catastrophe to a manageable chronic condition as shown by the global decline in the HIV incidence from 0.06% down to 0.019% and in Zimbabwe from 0.48% to 0.21% from 2011 to 2021 respectively. Improved access to ART, HIV prevention activities, HIV care and support services, has increased survival rates among people living with HIV and reduced HIV-related mortality, with an estimated 59,573 deaths averted in Zimbabwe in 2022 alone [2].

The Zimbabwe National HIV and AIDS Strategic Plan (ZNASP) 2021−2025 aims to achieve the 95-95-95 targets: 95% of people living with HIV knowing their status, 95% of those aware of their status being on sustained ART, and 95% of those receiving ART achieving viral suppression. The expected outcome of the 95-95-95 targets is a reduction in HIV incidence among adults and adolescents by 60%, a decrease in new infections among children to fewer than 50 cases per 100,000, lowering HIV-related mortality by 70%, and reducing stigma and discrimination among people living with HIV to less than 10% by 2026. Despite this progress, the provision of ART continues to pose significant costs and logistical challenges for healthcare systems, particularly in resource-limited settings. These difficulties are further compounded by the recent reductions in global health financing, with Africa experiencing notable aid cuts [3,4]. This threatens the hard-won gains toward achieving the 95-95-95 targets.

While numerous studies have examined the economic impact of HIV care in various sub-Saharan African settings, most research has focused on the patient-side financial burden [4–9]. Less attention has been paid to quantifying the provider-side costs of delivering these essential services, particularly in resource-constrained environments like Zimbabwe that continue to experience high HIV prevalence [10]. Provider cost analyses have typically relied on historical allocation methods that distribute resources based on past budgetary patterns rather than actual resource consumption. Time-driven activity-based costing offers superior transparency by documenting itemized costs across the entire treatment pathway based on actual resource utilization [10–12]. This approach is crucial for identifying technical efficiencies and ensuring long-term program sustainability.

In the healthcare sector, traditional costing and budgeting methods often rely on historical data, leading to challenges such as information asymmetry. On the other hand, value-based costing methods, including Time-Driven Activity-Based Costing (TDABC), developed by Kaplan et al., consider the resources and time required for each step in a process [13]. In healthcare, TDABC offers valuable insights into resource capacity and utilization, addressing issues like shared resources and task-shifting to enhance efficiency. This study aimed to measure the provider cost of HIV treatment in Zimbabwe using TDABC. Time-driven activity-based costing provides costs associated with ART service delivery cost drivers and potential areas of costs optimization especially in resource limited settings.

## Methods and materials

A cross-sectional design was used. The TDABC model as described by Kaplan and Anderson was used to cost HIV treatment in eleven (11) health facilities in Zimbabwe purposively sampled to ensure regional representation and inclusivity [13].

### Study setting

Zimbabwe is a landlocked country in the southern region of the Sub-Saharan Africa. It has a total of ten administrative provinces and sixty-three health districts. The country boasts of a high literacy rate, is multi-lingual and has a vibrant tourism industry housing one of the seven wonders of the world Victoria Falls, locally called Mosia-o-Tunya. Health services in Zimbabwe are delivered through a primary healthcare approach, established in the early 1980s. It is a lower-middle income country with a gross national income of USD3,220.00 per capita purchase power parity and a gross domestic product (GDP) of USD 1,773.90 in 2021. The diaspora remittances is the major foreign currency earner, remitting about one billion each year. The country’s current health expenditure was USD60.74, lower than the regional average of USD84.29.

Zimbabwe is conflicted by double burden of communicable and non-communicable diseases. Economic challenges have impacted funding for HIV treatment and care, whose domestic funding is primarily through the National AIDS Trust Fund (NATF), which allocates about 50% of its budget for ARV procurement. Funding for HIV treatment and prevention largely comes from partners like the Global Fund and the United States President’s Emergency Plan for AIDS Relief (PEPFAR). Services are available at all levels of care, including primary health facilities and various hospital tiers.

### Study population, sampling and sample size determination

The study analyzed presumptive, confirmed, and notified HIV cases, treatment-related ROCs, and healthcare workers from eleven sites, with a minimum target sample size of 4,422 for provider-ROC observations to ensure data saturation. Eleven health facilities were purposefully selected in consultation with MoHCC and partners. Selected facilities were high volume sites, sample of rural and urban, sample of clinics, districts, provinces and central hospitals level, ART treatment outcomes and one ART specialized private clinic.

### Development of process maps

A multi-stakeholder meeting with experts from the Ministry of Health and Child Care (MoHCC), partners and the University of Zimbabwe was conducted to develop ART process maps for both adult and paediatrics. Each process map outlines all tasks and resources required to deliver a service and delineates the activities required to provide patient care along the care pathway. The process maps were validated during the data collection to reflect the care processes existing in health facilities.

### Data collection

Data collection was done in 18 days starting from the 9^th^ to the 21^st^ of October in 2023. Teams of four qualified nurses recruited from non-HIV care settings were trained on the protocol to collect quality data. This reduced chances of inference during data collection. They were trained on protocol ethics, data collection methodologies which also covered the fundamentals of time-driven activity-based costing, interviewing techniques, data quality and use of the open data kit (ODK) technology.

Four facility-based data collection instruments were developed to map HIV services, healthcare cadres, equipment, materials, and laboratory consumables at health facilities. Paired provider-recipient-of-care pair sessions were recorded iteratively in minutes, and this included cadre providing the service, ROC details, materials used, medicines and physical space required to provide the care. Data points were collected at client registration, vital signs measurement, nurse consultations, HIV testing, and medical officer consultations, collection and processing of laboratory samples, collection of medicines from pharmacy and provision of health education. Data was stored in a central server, reviewed daily for quality and errors, and restricted to study team members. Team leaders participated in data cleaning and validation. Stata 18 and SPSS were used for data cleaning and costing mode.

### Costing

The cost at each stage of the ROC pathway was measured using the unit of time at the point of care provision. The estimated cost per minute was used to calculate the capacity cost rate of each of the domains (personnel, medicines and consumables, space and equipment, laboratory and indirect costs). The capacity cost rate (CCR) is a measure of a resource’s cost divided by its practical capacity, which is determined on a minute-by-minute basis. In addition, the resources required for the services at different healthcare levels were mapped and valued at the provider-ROC point level. To estimate the cost per patient in each delineated stage, we measured the practical capacity of the resources utilized to serve the patient at every stage. Practical capacity (*T*) is the total number of minutes of resource availability. Secondly, we calculated the capacity cost rate (*ccr*_*i*_) at every stage *i*, the resource cost divided by the practical capacity. Thirdly, the cost of providing a service to the ROC *j* at stage *i* per cost domain (*c*_*ij*_) was calculated as the product of capacity cost rate and time taken to serve the ROC at stage *i* (*m*_*ij*_), that is, *c_ij_*=ccr_i_*m_ij_. Indirect costs included water, support staff, administration costs and consumables whose cost estimates were calculated from facility-based consolidated budget expenditure estimates. Adding all the cost domains for all stages gave the provider cost per ROC.

To calculate the capacity cost rate for equipment, we utilized the most recent procurement data from the government and other health funders to obtain the cost of the equipment. The useful life of the equipment was estimated using information from procurement departments. For instance, furniture such as cabinets, tables, chairs, benches, and other equipment such as GeneXpert was assumed to be replaced after 10 years while computers and automobiles were replaced after 5 years. Other small tools like thermometers were thought to last for only a year. The replacement cost was then divided by the useful life span to obtain the annual replacement cost. Information on the practical capacity of the equipment was obtained during data collection from each facility. The capacity cost rate of equipment was then obtained by dividing the annual replacement cost by the annual number of minutes in which the equipment is utilized. An average time to provide care at each stage was used to estimate the cost of each stage. Literature was used to determine the average survival of each person starting antiretroviral therapy (ART), and HIV incidence and survival rate were used to estimate the total number of persons living with HIV. Space measurements were also used to estimate the current value of a building.

### Ethics approval and consent to participate

This study was approved by the Joint Review Ethics Committee (JREC/325/2023) and Medical Research Council of Zimbabwe (MRCZ), approval number MRCZ/A/3101 and Sally Mugabe Central Hospital Ethics Committee SMCHE021023/24. Completed tools, including written informed consent forms from service providers and recipients-of-care were sent to a central server where the data was checked for quality and errors on daily basis. Written consent was sought from parents or guardians for minors. This study complies with the World Medical Association (WMA)’s Declaration of Helsinki (https://www.wma.net/policies-post/wma-declaration-of-helsinki/) regarding ethical principles for medical research involving human participants, including research using identifiable human material or data.

## Results and discussion

All eleven reporting facilities provided HIV care and treatment services. A total of 2549 provider-ROC observations were made throughout the 68 observed stages. Adult ART had 28 stages and 793 provider-ROC observations, Paediatric ART had 17 stages and 451 ROC observations, 209 observations in six pharmacy stages and 393 observations in 17 laboratory stages.

### Annual cumulative costs of ART in Zimbabwe

In 2022, Zimbabwe provided antiretroviral therapy (ART) to 1,245,633 people living with HIV at a total annual provider cost of $168.66 million. This financial burden was predominantly shouldered by lower-level health facilities, which accounted for approximately 75% of the total costs due to the high volume of patients they served (Table 1). The national expenditure included $8.40 million for new first-line initiations and $14.61 million for initiating and maintaining patients on second-line regimens for the first year, with the majority ($145.65 million) allocated to maintaining existing stable patients on first-line regimens.

**Table 1:** Crude estimates of minimum annual ART cost, 2022.

Type of ROC	Central Hospitals	Provincial Hospitals	District Hospitals	Primary care Centers	Total
Number of new first-line ROC	1092	887	5093	29904	36976
Number of existing first-line ROC	25913	21639	178714	934734	1161000
Number of second-line ROC	6362	2009	13669	25617	47657
12 months cost for one new ROC on first-line ART	305	292	229	222	
12 months cost for one existing ROC on first-line ART	201	159	128	122	
12 month cost of one ROC on second-line ART	374	387	299	288	
Total ART costs for new ROCs on first line (US$)	332,668	258,596	1,164,038	6,641,036	8,396,338
Total ART costs for existing ROCs on first line (US$)	5,212,228	3,437,654	22,877,402	114,125,138	145,652,421
Total ART costs for ROCs during first year on Second line (US$)	2,382,326	777,137	4,087,885	7,366,783	14,614,130
Total provider costs by level of care	7,927,222	4,473,386	28,129,325	128,132,957	168,662,890

ART, antiretroviral therapy; ROC, recipient of care; US$, United States dollars.

When disaggregated by facility type, district hospitals incurred $28.13million, provincial hospitals $4.47 million, and central hospitals $7.93 million in ART-related costs (Table 1). Projections from 2023 to 2026 indicate that the total cost of providing ART will decline marginally from $202 million in 2023 to $192 million, representing a 5% decrease by 2026 (Table 2). This overall decrease is largely driven by a steady reduction in HIV incidence in children, based on the UNAIDS HIV estimates, while the number of adolescents and adults living with HIV is expected to remain relatively stable during this period.

**Table 2:** Adult and Adolescent ART Projections to 2026.

	Adult ROC (15 + years			Child ART ROC (0–14yrs)		
Year	Number of existing adult ART ROC	Estimated number of new ART ROC	Annual cost estimates	Number of children needing ART	Annual cost estimates	Total Cost (US$)
2023	1218983	13136	172,809,282	66,074	29,770,482	**202,579,764**
2024	1217246	13655	172,806,325	58,282	26,259,697	**199,066,022**
2025	1215723	14598	172,362,173	51,513	23,209,838	**195,572,011**
2026	1212925	13678	171,891,536	45,613	20,551,518	**192,443,054**

ART, antiretroviral therapy; ROC, recipient of care; US$, United States dollars.

*Source: HIV Estimates* [14].

### Provider cost of ART services

The average cost of initiating ART to an adolescent and/or adult was $57.05. Follow-up costs declined along the continuum of care, with six and twelve-month reviews averaging $36.97 and $33.88 respectively (Table 3). The cost of providing HIV care and treatment services during the first year for a new recipient of care (ROC) totaled $252.78, while maintaining a stable ROC on treatment averaged $138.93.

**Table 3:** Total average cost of adolescents and Adult HIV care and treatment (first line).

	Sally Mugabe	Mpilo	Bindura	Gwanda	Mt Darwin	Beitbridge	Newlands	Luveve	Mbare	Dotito	Zezani	Average	Range
New ROC First line													
Newly initiated on ART	71.92	85.44	53.11	65.05	42.69	50.32	66.67	64.49	53.50	33.54	40.78	57.05	33.54-85.44
Review after one month	34.10	37.68	28.09	31.67	25.98	25.63	32.34	27.41	28.94	19.80	24.02	28.70	19.80-37.68
Review after three months	33.75	38.07	28.18	31.58	25.46	25.60	32.60	27.51	28.52	20.09	21.92	28.48	20.09-38.07
Review after six months	56.42	65.26	80.63	66.57	52.15	55.38	40.51	56.14	52.88	45.56	50.92	56.58	40.51-80.63
Review after 9 months	33.75	38.07	28.18	31.58	25.46	25.60	32.60	27.51	28.52	20.09	21.92	28.48	20.09-38.07
Review after 12 months	52.64	62.20	76.90	61.58	50.23	52.61	38.04	53.63	50.61	43.44	46.58	53.50	38.04-76.90
Total cost of ART in first 12 months	282.57	326.71	295.07	288.01	221.95	235.16	242.78	256.70	242.94	182.54	206.13	252.78	182.53-326.71
Established ROC First line													
First review	52.64	62.20	76.90	61.58	50.23	52.61	38.04	53.63	50.61	43.44	46.58	53.50	38.04-76.90
Review after three months	33.75	38.07	28.18	31.58	25.46	25.60	32.60	27.51	28.52	20.09	21.92	28.48	20.09-38.07
Review after six months	33.75	38.07	28.18	31.58	25.46	25.60	32.60	27.51	28.52	20.09	21.92	28.48	20.09-38.07
Review after 9 months	33.75	38.07	28.18	31.58	25.46	25.60	32.60	27.51	28.52	20.09	21.92	28.48	20.09-38.07
Adult ART provider cost – existing 1st line ART ROC	153.88	176.41	161.43	156.30	126.59	129.43	135.86	136.17	136.15	103.72	112.33	138.93	103.72-176.41

ART, antiretroviral therapy; ROC, recipient of care; US$, United States dollars.

Initiation costs varied by facility, with lower costs at clinics ($33.54 at Dotito Rural Clinic), compared to hospitals ($85.44 at Mpilo). However, the total ART provision costs were comparable across hospitals and urban clinics. The six month review for new ROCs was the most expensive follow-up visit at $56.58. Switching to second line cost $78.68, with Mpilo remaining the most expensive facility for ART service provision at $114,07 for second-line therapy, while Dotito had the lowest cost at $49.72 (Table 4). The annual cost of maintaining a stable second line ROC averaged $174.93

**Table 4:** Total Average Cost of Adolescent and Adult HIV Care and Treatment (second-line).

	Sally Mugabe	Mpilo	Bindura	Gwanda	Mt Darwin	Beitbridge	Newlands	Luveve	Mbare	Dotito	Zezani	Average	Range
ART provider cost for second line (US$)													
Registration processes for EAC sessions	37.43	41.16	16.79	20.18	6.35	4.81	32.85	6.03	9.41	3.94	4.04	16.63	3.94-41.16
Enhanced Adherence Counselling sessions (3 as per OSDM)	3.48	2.64	14.25	3.81	3.39	3.15	4.86	2.10	0.90	0.66	4.14	3.94	0.66-14.25
Sample collection	1.26	1.86	1.99	1.31	1.72	1.44	1.20	1.30	0.93	0.95	0.96	1.36	0.93-1.99
Chemistry. TB and CRAG	16.26	25.65	21.13	24.14	17.13	19.29	17.37	27.05	20.39	15.49	18.77	20.24	15.49-27.05
Viral load	19.39	24.85	49.83	30.38	25.09	27.07	5.79	26.25	22.52	23.45	25.01	25.42	5.79-49.83
Initiate second treatment	2.02	3.29	3.48	1.86	1.94	1.55	1.52	0.66	0.29	0.31	1.03	1.63	0.29-3.48
Pharmacy with one month supply of 2nd line medicines	12.32	15.39	13.16	16.43	15.30	15.79	12.71	17.09	17.52	11.06	12.05	14.44	11.06-17.52
Total Average cost of Initiating ROC to second line ART	92.16	114.84	120.63	98.11	70.92	73.10	76.31	80.48	71.96	55.86	66.00	83.67	55.86-120.63
Total cost of ART first 12 months of switching to second line	347.81	401.12	407.59	366.06	295.18	302.95	297.42	317.69	306.40	249.85	276.35	324.40	249.85-407.59
Established ROC Second Line													
First review	61.64	71.20	85.90	70.58	59.23	61.61	47.04	62.63	59.61	52.44	55.58	62.50	47.04-85.9
Review after three months	42.75	47.07	37.18	40.58	34.46	34.60	41.60	36.51	37.52	29.09	30.92	37.48	29.09-47.07
Review after six months	42.75	47.07	37.18	40.58	34.46	34.60	41.60	36.51	37.52	29.09	30.92	37.48	29.09-47.07
Review after 9 months	42.75	47.07	37.18	40.58	34.46	34.60	41.60	36.51	37.52	29.09	30.92	37.48	29.09-47.07
Adult ART provider cost – existing 2nd line ART ROC	189.88	212.41	197.43	192.30	162.59	165.43	171.86	172.17	172.15	139.72	148.33	174.93	47.04-85.90

For children under two years, ART initiation averaged $62.70, with 1–5 month and 7–11 month follow-ups costing $135.85 and $134.75, respectively (Table 5). The national average annual cost for the paediatric ART was $450.56, with clinics generally incurring lower costs than higher-level facilities. Among the clinics, Luveve had the highest paediatric ART provider costs.

**Table 5:** Provider Costs of Providing Paediatric HIV Care and Treatment Services.

	Sally Mugabe	Mpilo	Bindura	Gwanda	Mt Darwin	Beitbridge	Newlands	Luveve	Mbare	Dotito	Zezani	Average
												
Newly initiated child	73.24	85.05	66.08	69.68	51.06	55.77	75.05	64.66	57.98	42.41	48.72	62.70
Community/telehealth review	2.41	2.17	2.32	2.01	2.08	1.23	1.21	1.21	1.44	0.41	2.90	1.76
Provider cost of 1–5 months reviews	129.20	144.85	133.90	147.15	139.95	139.30	128.81	147.47	141.10	106.75	135.90	135.85
Provider cost for 6 months review	56.31	66.84	94.87	72.12	53.38	65.64	63.91	66.48	55.78	45.84	52.92	63.10
Provider cost of 7–11 months	128.75	143.25	135.85	148.70	133.00	137.45	127.11	147.87	138.10	109.35	132.85	134.75
Provider cost of the review in 12 months	45.22	53.61	75.77	60.04	51.58	55.20	31.35	56.17	49.85	45.35	52.19	52.39
Annual provider cost	435.13	495.77	508.79	499.70	431.05	454.59	427.45	483.87	444.25	350.11	425.48	450.56

HIV, human immunodeficiency virus.

### Cost drivers for ART in Zimbabwe

**Fig 1:**
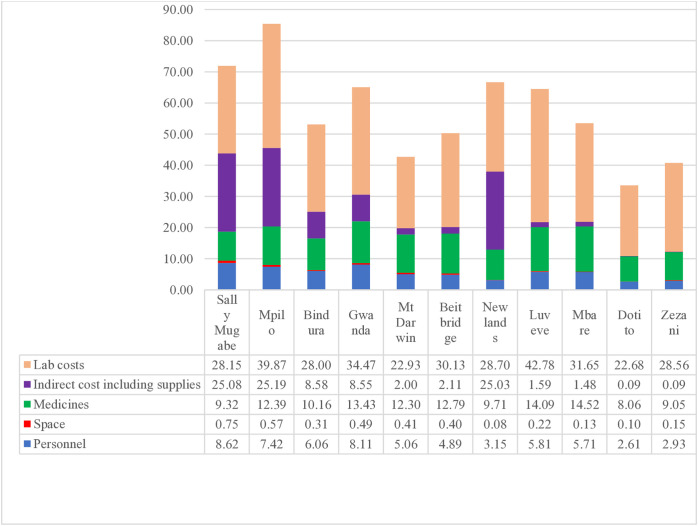
Annual provider cost drivers of ART adult first line ROC.

The primary cost drivers for adult ART were laboratory services at $30.72, followed by medicines at $11.44 and indirect costs at $9.07 (Fig 1). For paediatric ART, the cost structure differed with medicine costs being the highest at $27.98, followed by laboratory costs at $22.43 and indirect costs at $7.56.

**Fig 2:**
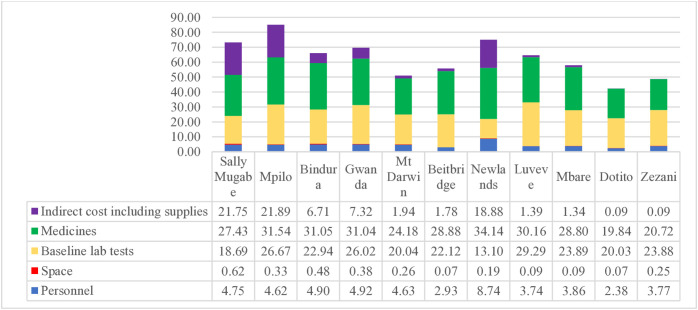
Cost domains of annual provider cost for ART for children less than 2 years of age.

As illustrated in Fig 1, costs drivers for antiretroviral therapy in adults were mainly laboratory, $30.72 followed by medicines, $11.44 and indirect costs, $9.07. In paediatric ART (Fig 2), medicine costs were the major driver of ART, $27.98 followed by laboratory costs, $22.43 and indirect costs, $7.56.

### Service provision of HIV treatment services and unit costs

A total of 159 health care providers interviewed distributed across eight (8) provider profiles: nurse/HIV focal person, primary counsellor (PC), medical officer, nurse aid, data clerk, community volunteer, reception manager and microscopist as shown in Table 6. At the central hospitals, medical officers conducted the initial ART assessment and continued all follow-up reviews through the first six months of treatment initiation. In contrast, at other facilities ranging from provincial level down to clinics, nurses provided nearly all ART services with support from PCs for HIV testing. Similar staffing patterns were observed in paediatric ART service delivery.

**Table 6:** Health Care Worker Providing Adult ART Services and Unit Cost, Zimbabwe TDABC Study, 2023.

Stage name		Cost per facility in US$													
		Sally Mugabe		Mpilo	Bindura	Gwanda	Mt Darwin		Beitbridge	Newlands	Luveve	Mbare	Dotito	Zezani	
Record pulling		0.12		0.18	0.26	0.24	0.31		0.31	0.13	0.02	0.24	0.10	0.31	
Registration		0.40		0.22	0.49	0.49	0.25		0.11	0.18	0.06	0.29	0.10	0.43	
Group education		1.42		2.81	1.09	2.02	0.60		0.07	0.30	0.91	1.57	0.93	0.35	
Vital signs		0.21		0.14	0.20	0.29	0.08		0.15	0.30	0.27	0.37	0.08	0.12	
Initial consultation		0.92		0.24	1.28	1.21	0.28		0.55	0.30	0.30	0.58	0.18	0.21	
Re-testing before ART		1.82		0.81	0.29	0.33	0.76		0.82	0.61	1.31	0.66	0.53	0.53	
ART assessment		1.57		0.77	0.44	0.88	0.12		0.52	0.40	1.05	0.55	0.12	0.12	
Physical examination		0.50		0.74	0.23	1.15	0.73		0.60	0.50	0.28	0.28	0.12	0.12	
Collection of blood for baseline tests		0.19		0.55	1.98	0.37	0.21		0.72	0.40	0.37	0.37	0.10	0.10	
Initiate ART		1.55		0.81	0.40	1.25	1.61		1.55	0.20	0.86	0.86	0.32	0.32	
Issue lab results		0.11		0.69	1.38	0.23	0.33		0.21	0.23	0.73	0.31	0.12	0.41	
Review after 1 month		0.83		1.47	1.56	0.73	1.05		0.55	2.73	1.02	0.66	0.98	0.13	
Review after 3 months		1.21		1.56	1.22	0.65	0.57		0.52	0.67	1.27	0.76	0.57	0.41	
Review every six months for viral load testing		0.34		1.36	0.17	0.61	0.31		0.50	2.70	0.76	0.51	0.77	0.44	
Review after 12 months		0.50		0.50	0.71	0.27	0.26		0.46	0.33	0.92	0.63	0.14	0.31	
Adherence counselling (2nd line)		0.33		3.98	0.62	0.66	0.84		0.56	0.73	1.15	0.25	0.22	0.12	
Review after 3 months post adherence counselling		0.74		0.77	0.96	0.32	1.00		0.80	0.80	0.57	0.54	0.69	0.69	
Lab assessment before initiating second-line treatment		1.29		1.34	0.72	0.73	1.38		0.93	0.31	0.73	0.84	0.83	0.83	
Initiate second treatment		2.70		2.80	1.48	1.26	1.59		1.03	0.38	1.05	0.21	0.21	0.21	
Review after 6 months for viral load testing		1.14		1.18	0.76	0.57	0.24		0.71	0.35	0.63	0.66	0.13	0.52	
Adherence counselling (3rd line)		0.12		0.11	0.62	1.07	0.84		0.72	5.10	0.66	0.70	0.22	0.22	
Comorbidity Screening (enrolment)		1.26		0.24	0.43	0.44	0.19		0.43	0.45	0.31	0.77	0.75	0.62	
Setting up DSD models		0.24		0.13	1.38	1.47	0.26		0.26		0.52	0.52	0.52	0.52	
DSD Registration		0.56		0.56	0.71	0.76	0.21		0.21		0.44	0.44	0.43	0.31	
DSD Consultation		0.81		0.31	0.62	0.66	0.61		0.60		0.31	0.31	0.41	0.83	
Group models – refill		0.52		0.20	0.69	0.73	0.63		0.62		0.42	0.42	0.80	1.14	
Individual Models – refill		0.44		0.44	0.49	0.52	0.56		0.34	0.20	0.66	0.66	0.41	0.52	
**Total**		**21.85**		**24.93**	**21.19**	**19.94**	**15.81**		**14.85**	**18.30**	**17.61**	**14.97**	**10.80**	**10.84**	
											
**KEY**	**Nurse**		**Nurse Aide**		**Medical Doctor**		**Primary Counsellor**		**Data Clerk**		**Community Volunteer**		**Reception Manager**		

Roles are distinguished by shading as follows: Nurse (green), Nurse Aide (peach), Medical Doctor (cyan), Primary Counsellor (pink), Data Clerk (yellow), Community Volunteer (brown), and Reception Manager (gray).

Labour costs, representing the expense of providing HIV care and treatment services of one newly diagnosed client through a complete consultation from registration to treatment initiation, were highest for the central hospitals, with Mpilo contributing $24.93 per patient. Clinics demonstrated lower labour costs, such as Dotito at $10.80 per patient. For paediatric HIV care and treatment, labour costs per patient were highest at the Newlands Specialist Clinic at $26.34 and the lowest was Dotito clinic at $8.10. Unlike adult services were facility level appeared to influence costs, paediatric ART service provision showed no distinct disparities by either level of care or geographic location.

### HIV care and treatment pathways

**Fig 3:**
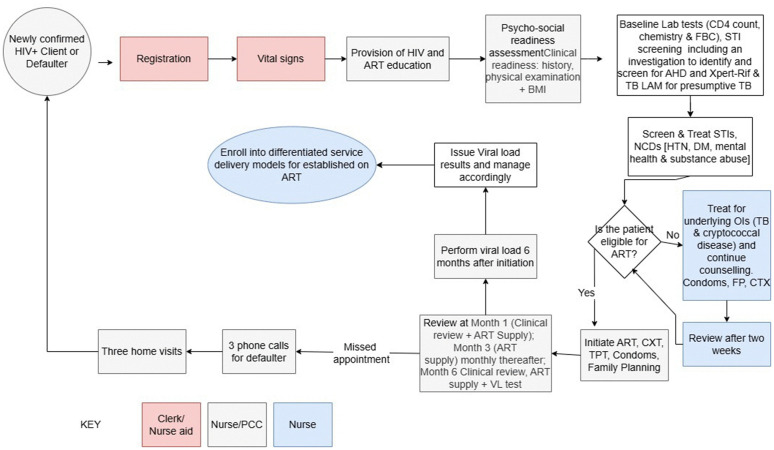
Adult ART pathway, MoHCC, 2023.

**Fig 4:**
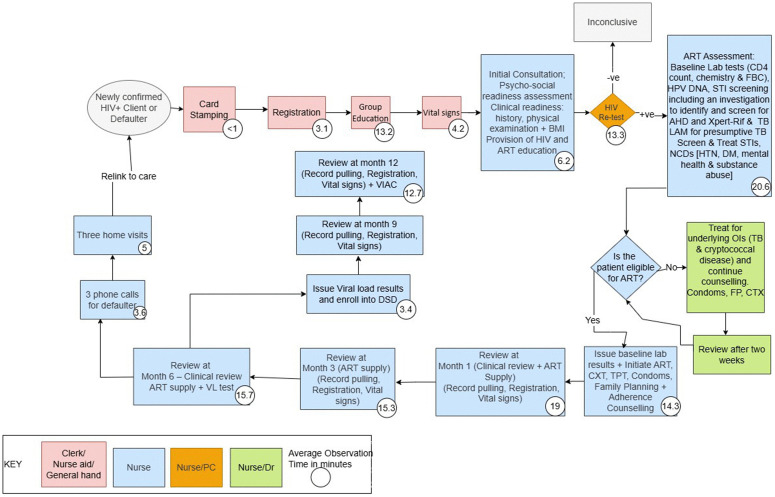
Adult ART pathway, Zimbabwe HIV TDABC, 2023. VL = Viral load; CXt = Cotrimoxazole; TPT = Tuberculosis Preventive Therapy; DSD = Differentiated Service Delivery; VIAC = Visual Inspection with Acetic Acid and Cervicography; OIs = Opportunistic Infections; FP = Family.

Patient registration and vital signs measurements were the first stages in adult ART service provision. During the full ART treatment cycle, assessment for ART initiation took the longest average time, 20.6 minutes. Group refill took one and a half times longer than individual refill options. There were no differences between the adult ART treatment pathway developed by national level experts (Fig 3) compared to the ART treatment pathway observed during data collection (Fig 4). Time taken for the review periods showed that the one month review period was the longest, 19 minutes, compared to the other subsequent review stages at 3, 6, 9 and 12 months which averaged 15 minutes.

There were no observed differences between Paediatric ART services from the OSDM recommended pathway to what was observed in the costing survey. There were no observed paediatric cases that were on second line treatment in the sampled provider-ROC pairs in this survey.

### Laboratory and pharmacy services

Although the laboratory and pharmacy services were not direct interventions as described in this survey, these were critical sub-pathways of the ART intervention. To facilitate proper costing and further description of these sub-pathways, they were costed separately. In the survey, each test was observed and costed separately (S1 Fig). The average time for sample transportation was 49.5 minutes with rural health facilities reporting longer times and lower number of results received. Viral load testing took the longest, 157 minutes, followed by nucleic acid testing with Gene Xpert, 63 minutes. S2 Fig in the supplementary material shows the pharmacy sub-pathway of the adult ART treatment pathway. Time taken for the different pharmacy stages were short, average 2.5 minutes.

This study provides a detailed cost analysis of HIV care and treatment services in Zimbabwe using TDABC. Experiences from successful previous TDABC for costing tuberculosis diagnosis and treatment in Zimbabwe and for HIV ART in Southern Africa were used to perfect the Zimbabwe ART costing study [15–17]. Previous studies in Zimbabwe and the Southern African region showed that $25.52 was required to provide ART to one client, lower than our study findings of $57.05 [18]. The difference likely reflects TDABC’s more comprehensive methodology, which captures the full resource consumption variations across different internal flows rather than using averaged historical allocations.

In this study, labor costs differed markedly by facility, with central hospitals such as Mpilo incurring higher costs of $24.93 per ROC compared to clinics such as Dotito at $10.80 per ROC. This pattern reflects differences in task shifting staffing models across facility levels. Task-shifting approach demonstrated that it was more cost effective to use nurses than medical officers without an effect on treatment outcome. This is consistent with previous studies which reported that task shifting from medical officer led HIV prevention, treatment and care to a nurse led care model has been reported in Africa and outside with no change in treatment outcomes [19,20].

In some settings, there were encouraging clinical outcomes with task shifting to nurse-led community HIV treatment models [21]. With persistent shortages of skilled health workers in many SSA countries, task shifting remains a viable solution to maintaining service continuity [22,23]. However, it should be noted labour costs during this costing exercise were artificially low due to the ongoing macroeconomic crisis in Zimbabwe, which has led to sub-optimal compensation for health workers [24].

The laboratory cost due to transportation of samples and cost of test kits were driving the cost of ART services in Zimbabwe. Studies from Tanzania and Mozambique used similar activity-based costing methods, and their costs were lower for stable patients but higher for unstable patients. In Tanzania facilities that had better coverage of services had higher provider costs compared facilities from low coverage of services. Similarly, one of the facilities sampled for this study was a private for non-profit institution providing state of the art services and it was spending more to treat HIV [25]. The high cost drivers from the laboratory services, medicines and indirect costs suggest that efforts to reduce costs should focus on optimizing laboratory testing algorithms and improving supply chain efficiency particularly for paediatric formulations which appeared disproportionately expensive. The declining follow-up costs along the continuum of care after a ROC has been initiated is consistent with the decrease of clinical monitoring required as a ROC stabilizes on treatment and with other studies in sub-Saharan Africa [9]. However, the substantial difference between first-line initiation compared to switching to second line costs highlights the financial importance of adherence and retention. Second line therapy was more resource intensive with initial costing $83.67 and annual maintenance costs averaging $174,93. Preventing treatment interruptions is therefore essential to minimize these high-cost transitions.

The annual cost of treating one HIV positive adult with first line medicines in Zimbabwe was US$230.78 which was lower than that of other lower-middle income countries, but higher than the sub-regional estimate of US$149 [17,26]. The higher initiation costs at central hospitals compared to clinics reflects the differences in staffing, infrastructure and patient complexity. This aligns with prior research showing that tertiary facilities incur higher unit costs due to specialized care and higher indirect costs [25]. Treatment monitoring tests like viral load testing have remained highly centralized. Resource constraints associated with centralization of viral load testing were a major barrier retarding the decentralizing HIV care [27]. As point of care viral load testing services become available, it is recommended that point of care viral load be further decentralized beyond PMTCT and adolescent clinics to allow decentralized ART services in Zimbabwe. Evidence show that there is no difference in treatment outcome in HIV patients receiving ART monitoring at primary care compared to centralized care [28].

Paediatric ART costs were notably higher than adult costs, with annual expenditure averaging $450.56 per child. This reflects the more intensive monitoring required for children, specialized formulations and more frequent clinical evaluations. These higher costs must be considered when planning comprehensive paediatric HIV services, especially in settings where children might be underrepresented in treatment programmes. Costs for Pediatric ART services were the highest, $590.83 and this was mainly due to laboratory, ART medicines and human resources. A similar study from Ethiopia paid less for ART in Paediatrics compared to the Zimbabwean study [29]. In other settings, costs of Paediatric ART were more expensive than what was observed in this study, $830 [30]. However, the cost drivers were similar, laboratory and ART medicines.

### Projected funding needs and optimization opportunities

The AIDS and TB programme estimates that the total population of people living with HIV will remain almost constant due to reduced HIV incidence and mortality. Although this will result in reduction of new cases, the total number of people living with HIV requiring treatment will remain relatively high resulting in increased costs of care. The increase in cost of care among HIV positive people was believed to be due to high incidence of chronic diseases and costs of medicines [31]. Despite the relatively stable population of people living with HIV in Zimbabwe, the projected annual costs for providing antiretroviral therapy remained substantial at $184.7 million for 2024 and $184.2 million for 2025, with adult ART services comprising the vast majority ($183.2 million and $182.8 million respectively) compared to pediatric ART ($1.55 million and $1.37 million). Available funding from the Government of Zimbabwe and partners fell significantly short, creating a $59.97 million gap in 2024 and a $3.72 million gap in 2025. With the aid cut, the funding gaps will increase substantially. To address these imbalances, cost reduction strategies such as optimizing laboratory monitoring protocols, modifying testing algorithms, and strategically evaluating and redirecting resources to prevention programmes could help sustain essential treatment services without compromising overall HIV program objectives.

This study reveals substantial inter-facility variation in provider time for identical ART care stages, indicating that internal processes are a major cost driver. By synthesizing the most efficient observed times into optimized care pathways, we estimate potential provider savings of approximately 25% for paediatric ART (reducing the mean first-year cost from $450.56 to $336.11 per child) and an 18% for a stable adult during the first year of initiation on ART. These efficiencies are already being realized in high-performing facilities such as Dotito (paediatric and administrative processes) and Mt Darwin (for efficient clinical reviews). If scaled national, such optimization could close a meaningful portion of the identified funding gap by freeing existing resources within the care delivery system.

A systematic review showed varied costs of HIV treatment depending on the economic status of the country. Low income countries were paying less to treat an HIV positive person with antiretroviral therapy (ART), US$789 per client per year compared to US$1,454 in high income countries [32]. This may be because of task shifting which was common in low-income countries compared to high income countries. Whilst the cost of medicines was driving high costs in low-income countries, in high income countries it was the cost of labour instead.

### Limitations

Although purposive sampling of health facilities was done to ensure maximum pathways were accounted for, it may not capture all variations in service delivery models throughout Zimbabwe. A related limitation is that our analysis of variability is conducted at facility level. The reported ranges describe dispersion between the mean values of the purposively sampled facilities and should not be interpreted as estimates of the total patient-level variance within the health system. Community-based activities had minimal representation and findings are reflective of interventions that are facility based. Analysis is from a provider perspective and a patient perspective will need to be considered in future studies given the importance of patient cost implications to treatment adherence and outcomes. Finally, costing was conducted during a specific time period and may not reflect seasonal variations in service delivery or costs.

## Conclusions

This TDABC study provides actionable evidence to inform HIV treatment and care programme sustainability in Zimbabwe and similar high-prevelance LMICs. The cost variations across facility types and patient populations reveal critical entry points for efficiency gains through differentiated service delivery and resource pooling. Notably, the higher costs of second-line therapies underscore the urgency of strengthening adherence programs and resistance surveillance to curb avoidable financial strain. The variations in cost drivers between adult and pediatric services highlight the need for tailored approaches to cost containment strategies for programme optimization.

While the projected decline in costs from $202 million to $192 million by 2026 reflect progress in reducing paediatric HIV incidence, it also underscores the continued financial requirements for sustained treatment at scale. Additionally, the substantial difference between first and second-line therapy costs emphasizes the importance of adherence support and resistance monitoring to maintain patients on less expensive regimens when clinically appropriate. As Zimbabwe transitions from donor-driven funding to domestic financing, these granular cost estimates serve as essential tools for strategic resource allocation, decentralized service optimization, and health system strengthening.

Recommended future direction is to implement operational studies at benchmark sites to codify best practices, followed by structured quality improvement initiatives to disseminate efficient workflows. Development of these efficient minimum packages of provider activities into national guidelines could enhance the financial sustainability of the HIV programme without compromising clinical objectives.

Future research should explore the relationship between costs and quality of care, the cost-effectiveness of differentiated service delivery models, the potential of digital health solutions for scalable efficiencies, and evaluate decentralized laboratory models to ensure cost containment without compromising care standards. Zimbabwe’s experience provides valuable lessons for countries navigating fiscal constraints while striving to deliver equitable and sustained HIV treatment.

## Supporting information

S1 Fig Laboratory average times, HIV TDABC Zimbabwe, 2023. (JPG)

S2 Fig Pharmacy process map, HIV TDABC Zimbabwe, 2023. (JPG)
