# Supplementary figures and images for: Provider costs of Antiretroviral therapy (ART) in Zimbabwe: The value of using time-driven activity based costing methods in a low resource setting
Source: PLoS One. 2026 Mar 25;21(3):e0345316. doi: 10.1371/journal.pone.0345316 (PMC13016285; doi:10.1371/journal.pone.0345316)

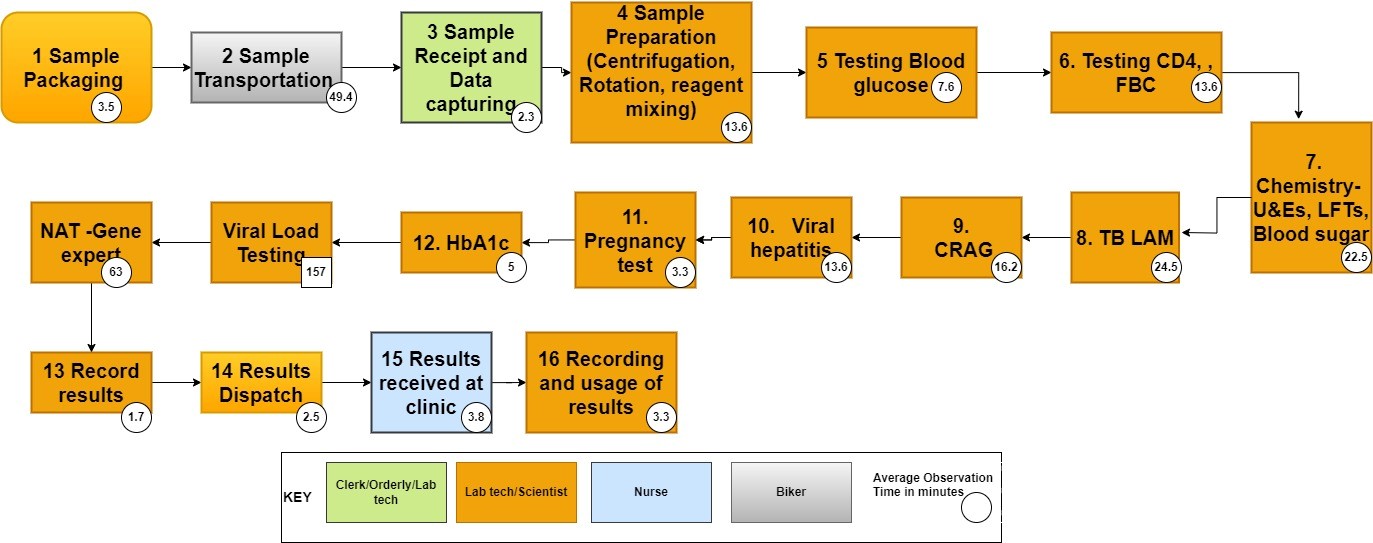

Supplement: S1 Fig — (JPG) [file pone.0345316.s001.jpg]

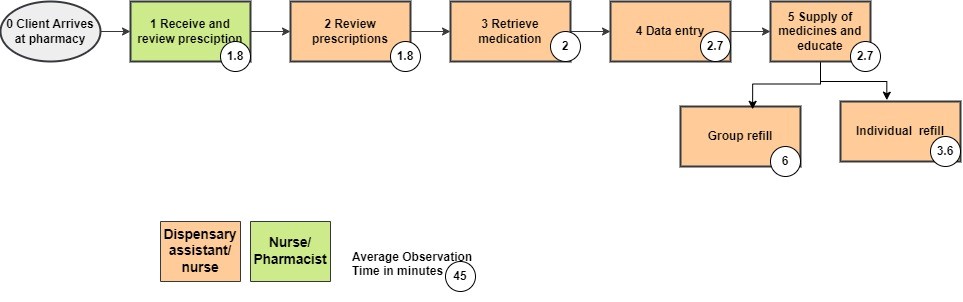

Supplement: S2 Fig — (JPG) [file pone.0345316.s002.jpg]
